# Visual Implicit Learning Abilities in Infants at Familial Risk for Language and Learning Impairments

**DOI:** 10.3390/ijerph19031877

**Published:** 2022-02-08

**Authors:** Roberta Bettoni, Chiara Cantiani, Valentina Riva, Massimo Molteni, Viola Macchi Cassia, Hermann Bulf

**Affiliations:** 1 Department of Psychology, Università degli Studi di Milano-Bicocca, 20126 Milano, Italy; viola.macchicassia@unimib.it (V.M.C.); hermann.bulf@unimib.it (H.B.); 2 Child Psychopathology Unit, Scientific Institute Eugenio Medea, 23842 Bosisio Parini, Italy; chiara.cantiani@lanostrafamiglia.it (C.C.); valentina.riva@lanostrafamiglia.it (V.R.); massimo.molteni@lanostrafamiglia.it (M.M.)

**Keywords:** statistical learning, rule learning, early markers, language learning impairment, infancy

## Abstract

The ability of infants to track transitional probabilities (Statistical Learning—SL) and to extract and generalize high-order rules (Rule Learning—RL) from sequences of items have been proposed as being pivotal for the acquisition of language and reading skills. Although there is ample evidence of specific associations between SL and RL abilities and, respectively, vocabulary and grammar skills, research exploring SL and RL as early markers of language and learning (dis)abilities is still scarce. Here we investigated the efficiency of visual SL and RL skills in typically developing (TD) seven-month-old infants and in seven-month-old infants at high risk (HR) for language learning impairment. Infants were tested in two visual-habituation tasks aimed to measure their ability to extract transitional probabilities (SL task) or high-order, repetition-based rules (RL task) from sequences of visual shapes. Post-habituation looking time preferences revealed that both TD and HR infants succeeded in learning the statistical structure (SL task), while only TD infants, but not HR infants, were able to learn and generalize the high-order rule (RL task). These findings suggest that SL and RL may contribute differently to the emergence of language learning impairment and support the hypothesis that a mechanism linked to the extraction of grammar structures may contribute to the disorder.

## 1. Introduction

Mastery of language can be difficult for some children. Indeed, around 7% of preschool children experience difficulties with expressive and/or receptive oral language and receive a diagnosis of developmental language disorder [1,2]. After school entry, language difficulties may also involve written language, leading to significant impairments in reading skills, also known as developmental dyslexia [3,4,5]. Several studies have shown that developmental language disorders and developmental dyslexia are often characterized by substantial overlap and heterogeneity of reading and linguistic impairments, and thus cannot always be differentiated (see e.g., [6]). Similarities of language phenotypes and comorbidity of language and reading problems [7] suggest that developmental language disorders and developmental dyslexia share common risk factors at either biological, neurocognitive, or environmental levels. For these reasons, the term ‘language learning impairment’ is widely used to encompass difficulties with expressive and/or receptive oral language and reading disabilities [8].

Although developmental language disorders is usually diagnosed around the age of four [2], and developmental dyslexia can only be diagnosed after school entry, early risk signs of these conditions can arise in the first years of life. Infants approach language acquisition with a set of early basic-level cognitive skills that have cascading effects on language development [9]. Among these early skills, statistical learning (SL) and rule learning (RL) are of particular relevance, as they support infants’ discovery of patterns and structures embedded in natural language.

SL refers to the ability to detect and encode structural relations defined by statistical regularities within item sequences. This ability is recognized as one of those supporting infants’ early word segmentation skills [10,11,12], and lexical development [13,14]. Indeed, when infants face natural speech streams, there are no reliable acoustic cues indicating word boundaries. However, in any given language, syllables that are part of the same words are more likely to occur together compared to syllables that are part of different words. Thus, transitional probabilities between syllables provide infants with one important source of information about word boundaries (see [15] for a review), together with other cues like rhythm and prosody, e.g., [16,17].

RL refers to the ability to extract high-order, repetition-based rules from item sequences, and to generalize them to novel items [18,19]. This ability is recognized as critical in tuning early infants’ learning to the grammatical structure of language and to the emergence of syntactic skills [20,21,22,23]. Indeed, beyond syllable-level statistics, any natural language also includes word-level statistics, as words are sequentially combined in highly predictable ways defined by grammar. Thus, tracking the invariance of words’ positions within the sentences allows infants to infer grammar categories and create an abstract and flexible representation of the relationship between the categories (e.g., ABA, noun-verb-noun), in which the speech units can be continually replaced (e.g., [24]).

Both SL and RL are available from birth [25,26,27] and are domain-general in nature, as they operate on a broad range of auditory [12,18] and visual stimuli [28,29,30,31,32] and allow infants to create an abstract representation of the learnt structures [29,30]. Overall, these implicit/procedural learning mechanisms would provide a domain-general strategy for the acquisition of language-specific knowledge. Not by chance, recent evidence supporting the relevance of SL and RL mechanisms for language development comes from prospective studies with infants showing significant predictive associations between visual SL abilities at six and eight months and vocabulary size at 13 and 22 months [13,14], and between the ability to extract and generalize rule-like structures from sequences of visual items at eight months and grammatical skills at 24 months [20].

In light of the role that SL and RL appears to play in supporting perception and learning from the linguistic input, it has been proposed that dysfunctions of these mechanisms in the earliest stages of development may disrupt infants’ learning from linguistic experience (e.g., [33,34,35]). This hypothesis is rooted in the neuroconstructivist view of atypical development e.g., [36], where phenotypical differences in language and learning outcomes are traced back to the earliest stages of development, when perturbation in basic cognitive processes can cause normal development trajectory to deviate across seemingly unrelated domains. Within this framework, the declarative-procedural model by Ullman [34] proposes that deficits in the linguistic domain could be linked back to a domain-general non-linguistic learning deficit affecting implicit or procedural learning. Despite its relevance, this hypothesis has been rarely investigated in preverbal infants.

Several studies reported SL and RL difficulties in adults and children with diagnosis of developmental language disorders or developmental dyslexia. For example, 6–14-year-old children with developmental language disorders require more time than typically developing children to detect and learn the statistical relationships embedded in streams of speech sounds or tones [37,38]. Similarly, adults and 8–12-year-old children with developmental dyslexia fail to pick up statistical relationships from auditory and visual stimuli and to generalize familiar rule-like patterns to novel stimuli e.g., [39,40,41,42,43,44]. These findings suggest that atypical SL and/or RL skills might contribute to the lexical, syntactic, and reading deficits that characterize language learning impairment. However, they do not inform us about the possible role that atypical SL and/or RL skills may play in earlier stages of development when, well before the acquisition of speech production, infants are faced with the task of extracting acoustic cues from natural language and mapping them onto linguistic information.

In the current study, we aimed to extend available evidence of associations between SL and RL abilities and the development of language and literacy skills by testing preverbal, typically developing (TD) infants and infants at high risk (HR) of developing language learning impairment, where risk is defined by having at least one first-degree relative with a diagnosis of developmental language disorders and/or developmental dyslexia. Indeed, it is known that language-related disorders run in families [45] and that infants at familial risk have both higher genetic and environmental liability to be diagnosed for language learning impairment compared to children not-at risk [2,46,47]. Therefore, studying infants who have at least one parent or sibling with developmental language disorders and/or developmental dyslexia allows insight into the developmental trajectory of the disorder, and has often been used as a research strategy to study its underlying causes (e.g., [48]).

To the best of our knowledge, only one study has addressed this issue by assessing auditory SL skills in 12-month-old infants at familial risk (vs. not at risk) for developmental dyslexia [49]. Infants were habituated to strings of three-element artificial words containing dependencies of the type a-x-c, b-x-d, in which the first and third elements remained fixed, while x elements changed. At test, typically developing infants discriminated between familiar (i.e., corresponding to the training language) and unfamiliar strings, while family-risk infants did not, indicating deficient statistical learning. To date, no studies have investigated RL abilities in infants at family risk for language and learning disorders.

Here, infants’ SL and RL skills were tested in the visual modality to control for learning biases that may originate from infants’ perceptual expertise with linguistic sounds or from possible atypicalities in the processing of linguistic sounds associated with language learning impairment. Indeed, it is known that children with language learning impairment exhibit a deficit in verbal working memory e.g., [2,50] and infants at high risk for language learning impairment show atypical processing of speech and non-speech auditory stimuli [51,52,53,54]. Even if previous studies showed that implicit learning deficits in children with language learning impairment cannot be explained solely by dysfunctions in verbal working memory (e.g., [55]), it is still unknown whether atypical auditory processing may play a role in preventing infants from tracking patterns and structure embedded in the speech input.

Moreover, testing SL and RL abilities using visual stimuli allowed us to investigate the role of early domain-general learning abilities on later language acquisition [36,56]. As discussed above, children with developmental dyslexia are deficient in picking up regularities from both linguistic and visual stimuli (e.g., [42,43,44]), a finding supporting the hypothesis of a domain-general implicit learning deficit at the roots of language learning impairment. However, it is still unexplored whether preverbal infants at high risk for language learning impairment show a similar widespread impairment in the ability to track statistical regularities and extract high-order structures from item sequences.

In Study 1, a group of TD infants and a group of HR infants aged seven months were tested in a standard visual SL task (see [57]) in which, after habituation to a continuous stream of geometrical shapes containing a statistical structure, looking time preference for a novel random structure over the familiar one was measured as an index of discrimination. In Study 2, two groups of TD and HR infants aged eight months were tested in a visual RL task (see [58]), in which they had to extract a repetition-based, non-adjacent structure (i.e., ABA) from sequences of visual shapes, and generalize it to new shapes. The infants’ success at RL would have been indexed by a looking time preference for novel shapes organized into an ABB rule-governed pattern following habituation to ABA sequences. Importantly, a subsample of TD and HR infants were followed longitudinally at seven and eight months and provided within-subjects measures of their visual SL and RL abilities.

Our expectation was that typically developing infants would be able to discriminate between trained and untrained stimuli, resulting in a novelty effect in both tasks. If SL and RL are impaired in HR infants, they might not be able to track the statistical dependencies in the SL and/or the non-adjacent structure in the RL task, resulting in failure to discriminate between the familiar and the novel sequences at test. Otherwise, they might display a preference for the familiar test sequences, indicating that their implicit sequential learning skills are less efficient than those of their typically developing peers, thus leading to an incomplete representation of the trained statistical and/or rule-like structure during habituation.

## 2. Study 1—Visual Statistical Learning Task

### 2.1. Materials and Methods

#### 2.1.1. Participants

Twenty seven-month-old infants at familial risk of language learning impairment (mean age = 207.47 days, *SE* = 2.15 days; 11 males) were included in the HR group; five had a positive family history for developmental language disorders, 10 for developmental dyslexia, and five for both. Three additional infants were tested but excluded from the final sample due to fussiness (*n* = 1) or because their looking time during at least one of the test trials were 3 SD above the group mean (*n* = 2). The TD group included 21 seven-month-old infants (mean age = 215.57 days, *SE* = 4.35 days; 11 males); two more infants were tested but excluded from the final sample due to fussiness (*n* = 1) or because their looking time at the test exceeded 3 SD from the group’s mean (*n* = 1). All infants in the final sample had a gestational age of at least 37 weeks, a birthweight ≥ 2500 g, an APGAR score of at least 8 at 1 and 5 min, a Bayley cognitive score [59] of at least 7, and no certified diagnosis of intellectual deficiency, sensory or neurological disorders. Demographic, cognitive, and clinical characteristics of the two groups are reported in Table 1; the two groups of infants did not differ on any of these variables.

The HR group was selected on the basis of parental reading difficulties based on a two-step procedure after [51]. First, an interview was used to determine whether any of the infant’s first-degree relatives received a clinical diagnosis of developmental language disorders or developmental dyslexia, and parents filled in the ‘Adult dyslexia checklist’ (ADCL) [60] questionnaire, a widespread screening tool for adults with dyslexia (e.g., [61]), composed of twenty questions with dichotomous items (with Yes/No answers). Second, parents who scored more than 5 at the ADCL, thus reporting reading difficulties, were directly evaluated by a clinical psychologist using standardized tests assessing word and non-word reading [62], and text-reading [63]. Infants were assigned to the HR group if at least one first-degree relative: (1) had a certified diagnosis of developmental language disorders and/or developmental dyslexia or (2) reported reading difficulties (ADCL > 5) and performed at least two standard deviations (SD) below the population mean on the two reading tasks. Infants’ families were recruited from three hospitals within the Lecco and Monza Brianza area (Northern Italy). Parents gave their informed written consent for their infant’s participation. Ethical and Scientific Committees of the all institutes involved in the study approved the study protocol following the ethical standards of the 1964 Helsinki Declaration.

#### 2.1.2. Apparatus, Stimuli, and Procedure

All infants were tested in a sound-isolated and dark-silent cabin while seated in the parent’s lap. Stimuli were generated using E-prime 2.0 software (Psychological Software Tool, Pittsburgh, PA) and presented on a black background on a 21-inch monitor with a resolution of 1360 × 768 pixels. A video camera, positioned above the presentation monitor, recorded the infant’s face and sent a visual input to a TV monitor outside the cabin to allow the online coding of infants’ looking times by an experimenter who was blind to the stimuli presented. The image of the infant’s face was also recorded via a Mini-DV digital recorder for the purpose of offline coding.

**Figure 1:**
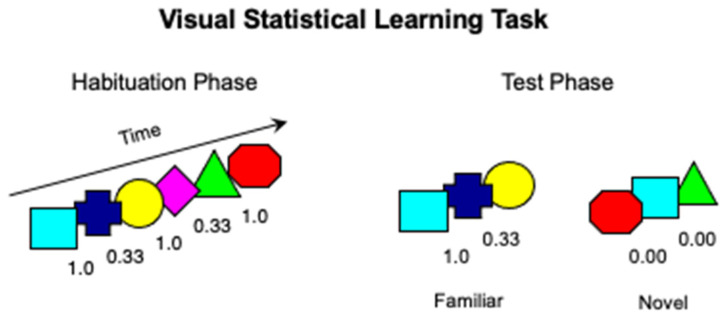
Schematic representation of the stimuli and procedure used in the visual Statistical Learning (SL) task (Study 1).

The stimuli and procedure were modeled after Bettoni et al. [64]. Infants were tested in an infant-controlled visual habituation task [65] in which they were presented with a continuous stream of 6 colored shapes (turquoise square, blue cross, yellow circle, pink diamond, green triangle, and red octagon) appearing one at a time in the center of the screen without breaks for 750 ms. Each shape loomed along both the vertical and horizontal axes ranging in size from 3 to 10 cm. In the habituation sequence, the six shapes were organized into three pairs, presented in a random order. This way, transitional probability between two consecutive shapes was 1.00 within pairs, and 0.33 between pairs. In the test sequence, the habituation sequence was presented with a novel sequence in which the six shapes were presented in a random order (transitional probability = 0), with the only constraint that no more than two identical shapes could appear in a row (Figure 1).

The habituation sequence was presented until the infant viewed 21 trials or met the habituation criterion (a 50% decline in looking time on three consecutive trials, relative to the total looking time on the first three trials). Each trial started with a cartoon animated image coupled with a sound appearing in the center of screen, which served as an attention-getter. As soon as the infant fixated on the screen, the cartoon was turned off and the first shape appeared. After habituation, infants viewed six test trials alternating between the habituation and the test sequences, with the order of presentation counterbalanced across participants. Each habituation and test trial was presented until the infant looked away from the screen for at least two consecutive seconds or accumulated a maximum looking time of 60 s.

The dependent variables for the habituation phase were online-coded looking times and number of trials to habituate, and the slope of the habituation looking times, which was calculated by fitting linear regression functions to each infant’s online-coded looking time (in seconds) across the habituation trials. The dependent variables analyzed for the test phase were online-coded looking times and proportional delta scores calculated as the difference between infant’s looking times to the novel and the familiar test trials, divided by total looking times to the two trials (i.e., novel − familiar/novel + familiar). For about half of the participants (*n* = 20), looking times during test trials were coded offline by a second independent observer who was blind to the stimuli presented. Inter-observer agreement between the two observers who coded the data live or from a digital recording, as computed on total fixation times on each of the six test trials, was r = 0.982 (*p* < 0.001, Pearson correlation).

At the end of the habituation task, the experimenter administered the cognitive subscale of the Bayley Scales of Infant Development [59].

### 2.2. Results

#### 2.2.1. Habituation Phase

Three independent sample *t*-tests revealed that the TD and HR groups did not differ in the total looking time, number of trials, and slope of looking times to habituate (see Table 2). The range of total looking times to habituate was 41.89–271.96 s for the TD group and 12.87–192.64 s for the HR group. An Analysis of Variance (ANOVA) with group (TD vs. HR) as the between-participants factor and habituation trials (first three vs. last three) as the within-participants factor confirmed the presence of an overall significant decline in mean looking times between the first three (*M* = 18.97 s, *SE* = 1.99) and the last three (*M* = 6.62 s, *SE* = 0.574) habituation trials, *F*(1,39) = 54.55, *p*  <  0.001, *ƞ^2^_p_* = 0.583. There was no significant main effect, *F*(1,39) = 1.172, *p* = 0.286, *ƞ^2^_p_* = 0.029, nor interaction, *F*(1,39) = 0.864, *p*  = 0 .358, *ƞ^2^_p_* = 0.022, involving the factor group.

#### 2.2.2. Test Phase

**Figure 2:**
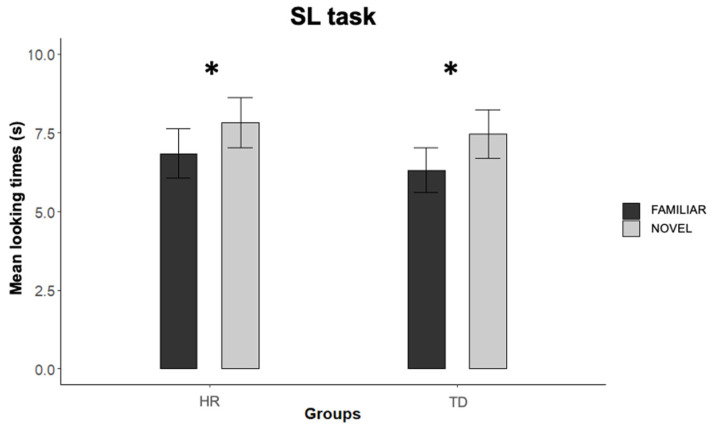
Mean looking times to the familiar and novel test trials for the HR infants and for the TD infants. Error bars represent standard error of the means; * *p* < 0.05.

To determine whether infants were able to discriminate between the familiar and novel sequences at test, the six test trials were grouped into three test trial pairs, each composed of a familiar and novel trial, and infants’ looking times to those trials were entered into an ANOVA with group (TD vs. HR) and test trial order (familiar first vs. novel first) as between-participants factors, and test trial pair (first vs. second vs. third) and test trial type (novel vs. familiar) as within-participants factors. The analysis revealed a main effect of test trial type, *F*(1,37) = 5.33; *p* = 0.027, *ƞ^2^_p_* = 0.126, with longer mean looking times to the novel sequences (*M* = 7.70 s; *SE* = 0.55, range = 2.29–13.96 s) compared to the familiar ones (*M* = 6.49 s; *SE* = 0.511, range = 1.63–16.82 s). There was no significant main effect, *F*(1,37) = 0.322; *p* > 0.574, *ƞ^2^_p_* = 0.009, or interactions, *F*s < 2.068, *p* > 0.159 involving the factor group (Figure 2).

Overall, results from both the habituation and test phase indicated that both TD and HR infants were equally able to extract the statistical structure embedded in the habituation sequence (Figure 2).

## 3. Study 2—Visual Rule Learning Task

### 3.1. Materials and Methods

#### 3.1.1. Participants

Nineteen 8-month-old infants (mean age = 247.05 days, *SE* = 3.90; 9 males) were included in the HR group (seven with positive family history for developmental language disorder, eight for developmental dyslexia, and four for both conditions), and 19 infants of the same age were included in the TD group (mean age = 247.68 days, *SE* = 2.24; 9 males). Six additional infants were tested and excluded from the final samples due to fussiness (HR group: *n* = 1, TD group: *n* = 1), technical error (TD group: *n* = 1) or because their looking time during at least one of the test trials exceeded 3 SD from the group’s mean (HR group: *n* = 2; TD group: *n* = 1).

Inclusion criteria were the same as those used in Study 1. Demographic, cognitive, and clinical characteristics of the HR and TD groups are reported in Table 3. The two groups did not differ on any of these variables except for the cognitive subscale of the Bayley Scales of Infant Development [59], for which both HR and TD infants’ scores were in the normal range, but the HR infants scored higher than the TD infants (but see Table 5 for the absence of group differences in the subsample of infants who participated in both Study 1 and 2).

Parents gave their informed written consent for their infant’s participation. Ethical and Scientific Committees of all institutes involved in the study approved the study protocol following the ethical standards of the 1964 Helsinki Declaration.

#### 3.1.2. Apparatus, Stimuli, and Procedure

**Figure 3:**
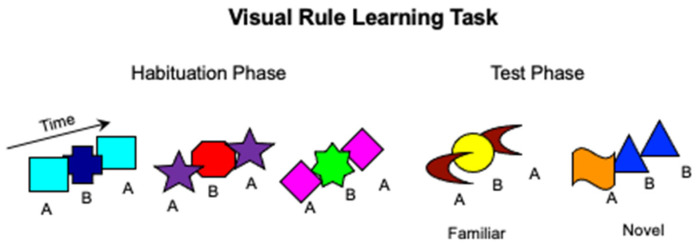
Schematic representation of the stimuli and procedure used in the visual Rule Learning (RL) task (Study 2).

The apparatus was the same as in Study 1, and the stimuli and procedure were modeled after Bulf et al. [58]. Infants were tested in an infant-controlled visual habituation procedure in which they were presented with ABA rule-based visual sequences composed by twelve colored geometrical shapes appearing one at a time at different spatial locations from left to right on the screen. Eight unique shapes were presented during the habituation phase, and four different unique shapes were shown during the test phase. Each shape was embedded in a virtual square of 10° × 10° visual angle when viewed from a distance of 60 cm. For the habituation triplets, four shapes were assigned to the A group, and four to the B group, and A and B images were randomly combined to obtain 16 different ABA triplets. Four novel shapes, two assigned to the A group and two assigned to the B group, were randomly combined to obtain four different ABA triplets (familiar rule) and four different ABB triplets (novel rule) (Figure 3). Within each triplet, shapes were presented sequentially from left to right: the first shape was displayed on the left side of the screen for 330 ms, the second was displayed in the middle of the screen for 330 ms, and the third shape was displayed on the right side of the screen for 830 ms. After the third shape disappeared, a 500 ms-blank screen was presented before the appearance of the next triplet.

Each trial started with a cartoon animated image coupled with a sound appearing in the center of screen, which was turned off as soon as the infant fixated on the screen, followed by the first image. Trials consisted of triads of images, presented in a random order, organized an ABA pattern, and continued until, after looking continuously for a minimum of 500 ms, the infant looked away for two consecutive seconds or looked for a maximum of 60 s. Infants were intended to be habituated when they viewed a maximum of 21 trials, or met the habituation criterion, which was defined as a 50% decline in looking time on three consecutive trials, relative to the looking time on the first three trials. Following habituation, infants viewed six test trials alternating between ABA and ABB triads. Test triplets were composed by images that differed from those shown during habituation to assess infants’ generalization of the familiar rule to new items. Test trial order (i.e., novel first or familiar first) was counterbalanced across participants. As in Study 1, the dependent variables for the habituation phase were online-coded looking times and number of trials to habituate, and the slope of the habituation looking times. The dependent variables analyzed for the test phase were online-coded looking times and proportional delta scores. For about half of the participants (*n* = 20), looking times during test trials were coded offline by a second independent observer who was blind to the stimuli presented. Inter-observer agreement between the two observers who coded the data live or from a digital recording, as computed on total fixation times on each of the six test trials, was r = 0.971 (*p* < 0.001, Pearson correlation).

At the end of the habituation task, the experimenter administered the Cognitive subscale of the Bayley Scales of Infant Development [59].

### 3.2. Results

#### 3.2.1. Habituation Phase

Independent samples *t*-tests performed on total looking time, number of trials, and the slope of looking times to habituate failed to show significant cross-group differences between the HR and TD infants (see Table 4). The range of habituation looking times was 15.82–150.90 s for the TD group, and 17.25–148.31 s for the HR group. A group (TD vs. HR) x habituation trials (first three vs. last three) ANOVA confirmed the presence of a significant decline in mean looking times between the first three (*M* = 10.32 s, *SE* = 1.05) and the last three (*M* = 4.37 s, *SE* = 0.42) habituation trials, *F*(1,36) = 78.643, *p*  <  0.001, *ƞ^2^_p_* = 0.686. There was no significant main effect, *F*(1,36) = 0.631, *p* = 0.432, *ƞ^2^_p_* = 0.017, nor interaction, *F*(1,36) = 0.104, *p* = 0.749, *ƞ^2^_p_* = 0.003, involving the factor group.

#### 3.2.2. Test Phase

TD and HR infants’ looking times to the familiar and the novel test triplets were compared by means of an ANOVA with group (TD vs. HR) and test trial order (familiar first vs. novel first) as between-participants factors, and test trial pair (first vs. second vs. third) and test trial type (novel vs. familiar) as within-participants factors. The analysis revealed a main effect of test trial pair, *F*(2,68) = 4.063, *p* = 0.022, *ƞ^2^_p_*  = 0.107, as infants looking times in the third trial pair (*M* = 4.71 s, *SE* = 0.43) were shorter than in the first (*M* = 6.17 s, *SE* = 0.61), *p* = 0.039, but not in the second (*M* = 5.91 s, *SE* = 0.54), *p* = 0.057, trial pairs. The ANOVA also revealed significant interactions between group, test order and test trial pair, *F*(2,68) = 8.224, *p* < 0.001, *ƞ^2^_p_*  = 0.195, and between group and test trial type, *F*(1,34) = 7.581, *p* = 0.009, *ƞ^2^_p_* = 0.182 (Figure 2). To follow-up these interactions, two separate 2 (test trial order) × 3 (test trial pair) × 2 (test trial type) ANOVAs were performed, one for each participants’ group. For the HR group, looking times did not differ between novel (*M* = 5.62 s, *SE* = 0.60, range = 2.20–10.98) and familiar (*M* = 5.04 s, *SE* = 0.74, range = 1.82–12.48) test trial types, *p >* 0.346. However, the analysis revealed a Test trial pair x Test trial order interaction, *F*(2,34) = 3.649, *p* = 0.037, *ƞ^2^_p_* = 0.177. Post-hoc comparisons (Bonferroni corrected *t*-tests) revealed that, when the test phase started with a novel trial, infants looked longer to the first (*M* = 15.46 s, *SE* = 3.05) test pair than to the second (*M* = 10.20 s, *SE* = 2.11), *p* > 0.014, and the third (*M* = 8.71 s, *SE* = 1.45), *p* < 0.03, pairs, while they did not change across pairs when the test phase started with a familiar trial (*M* = 9.37 s vs. 11.13 s vs. 9.12 s), *ps* > 0.327. Of note, however, test trial order did not impact infants’ looking time responses to the novel and familiar test trial, as the Test trial pair x Test trial order x Test trial type interaction was nonsignificant (*p* = 0.804), and no effects involving the test trial type factor were observed (*ps* > 0.346).

**Figure 4:**
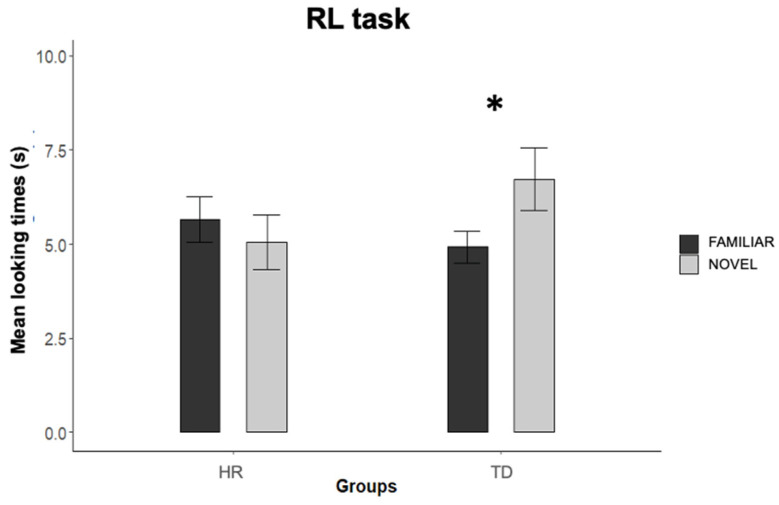
Mean looking times to the familiar and novel test trials for the HR infants and the TD infants. Error bars represent standard error of the means; * *p* < 0.05.

For the TD group, the analysis revealed a test trial type main effect, *F*(1,17) = 8.41, *p* = 0.010, *ƞ^2^_p_* = 0.331, as infants looked longer to the novel (*M* = 6.75 s, *SE* = 0.86, range = 3.34–16.07 s) than to the familiar (*M* = 4.97 s, *SE* = 0.43, range = 2.99–8.77 s) test sequence. There was also a Test trial pair x Test trial order interaction, *F*(2,34) = 4.751, *p* = 0.015, *ƞ^2^_p_* = 0.218, (Figure 4). Post-hoc comparisons (Bonferroni corrected *t*-tests) revealed that, when the test phase started with a familiar trial, infants looked longer to the first test pair (*M* = 14.49 s, *SE* = 1.45) than to the third one (*M* = 9.03 s, *SE* = 2.56), *p* < 0.02, while they did not change their looking times across pairs when the test phase started with a novel trial, *ps* > 0.142. However, test trial order did not impact infants’ looking time responses to the novel and familiar test trial, as no effects involving test trial order and the test trial type factor were observed (*p* = 0.820).

Overall, results suggested that, although the TD and the HR infants did not differ in their habituation performance to the ABA sequences, they did differ in their ability to generalize the ABA structure to the new items at test, as only the TD infants, but not the HR infants, discriminated the familiar triplets from the novel ones.

## 4. Within-Subjects Comparison of Infants’ Performance in the SL and RL Tasks

A subsample of 15 HR infants (eight males, four with a family history of developmental language disorder, seven for developmental dyslexia, four for both conditions) and 10 TD infants (five males) who participated in the visual SL task at seven months of age was followed longitudinally at eight months and also participated in the visual RL tasks. The two groups were homogeneous on all considered demographic, cognitive, and clinical variables (see Table 5). This gave us the opportunity to provide within-subject comparison of HR and TD infant performance across the two tasks.

**Figure 5:**
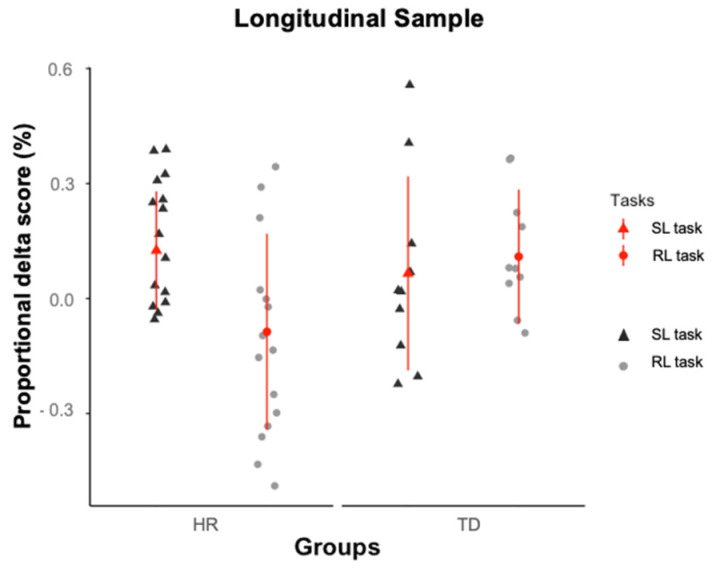
Individual data points of the proportional delta scores for the HR and the TD infants who participated in both SL and RL tasks. The red dots and lines represent, respectively, the group means and standard deviations.

To this end, proportional delta scores obtained from total looking times on the novel and familiar test triplets at test (Novel − Familiar/Novel + Familiar) were entered into a group (HR vs. TD) x task (SL vs. RL) ANOVA, which revealed the presence of a significant interaction between the two factors, *F*(1,23) = 8.650, *p* = 0.007, *ƞ^2^_p_* = 0.300, and no significant main effects (*ps* > 0.200). Follow-up *t*-tests revealed that the TD infants’ proportional delta scores did not differ across the SL (*M* = 0.029, *SE* = 0.186) and the RL tasks, (*M* = 0.109, *SE* = 0.175), *t*(9) = 0.960, *p* = 0.362, *d* = 0.304, while the HR infants showed a larger proportional delta scores in the SL task (*M* = 0.125, *SE* = 0.154) compared to the RL task (*M* = −0.087, *SE* = 0.255), *t*(14) = 3.593, *p* = 0.003, *d* = −0.928. Moreover, cross-groups comparisons of proportional delta scores were significant for the RL task, *t*(23) = 2.277 *p* = 0.032, *d* = 0.862, but not for the SL task, *t*(23) = 1.412, *p* = 0.468, *d* = −0.576 (Figure 5).

These findings confirm those obtained from the analyses performed on the full samples of HR and TD infants participating in Study 1 and Study 2, indicating that both HR and TD infants were sensitive to the statistical structure embedded in the sequence presented in the visual SL task, while only TD infants, and not HR infants, were able to generalize the rule-like structure embedded in the habituation sequence to novel items in the RL task.

## 5. Discussion

Several studies reported SL and RL difficulties in adults and children with developmental dyslexia and developmental language disorder that are often combined under the term language learning impairment [8,66], suggesting that an implicit and procedural learning deficit could contribute to language learning impairment [34].

The present study aimed to investigate, for the first time, whether early signs of domain-general implicit learning deficits are evident in the first year of life, before language acquisition, in infants who are at familial risk for language learning impairment by virtue of having a first degree relative with a diagnosis of developmental dyslexia or developmental language disorder. In order to determine whether domain-general ability to pick up regularities from the environment might be affected in HR infants, SL and RL abilities were tested using visual rather than acoustic stimuli. Previous studies showed that adults and children with language learning impairment perform poorer than their undiagnosed peers in catching patterns embedded in visual (e.g., [40,41,42,43,44]) and speech (e.g., [37,38,39]) streams, suggesting that language learning impairment is associated with a learning disadvantage that is not strictly related to the auditory and linguistic nature of the stimuli used. However, only one study has investigated implicit learning skills in infants at high-risk for developmental dyslexia using linguistic stimuli [49], leaving unanswered the question of whether a widespread domain-general deficit in detecting patterns and structure in the environment is present in HR infants.

The results failed to show differences in habituation performance between HR and TD infants in both the SL and the RL task, suggesting that learning occurred similarly for infants in the two groups. This finding was unexpected in light of earlier, conflicting evidence of differential habituation performance to auditory stimuli and linguistic sounds in infants at TD and HR for developmental dyslexia and language learning impairment. The amount of time infants need to habituate to a given stimulus is considered a reliable indicator of the amount of attentional resources allocated to the stimulus over time, and the efficiency of information processing [67]. In the case of non-speech auditory stimuli, Choudhury et al. [68] found that HR infants had shallow slopes and required more trials to habituate to unchanging sound pairs compared to TD infants, thus suggesting that learning occurred less efficiently in at-risk infants. In contrast, using a head-turn procedure, Kerkhoff et al. [49] reported shorter looking times in HR compared to TD infants during familiarization to speech sounds, suggesting that at-risk infants allocated less attentional resources to the processing of the familiarized stimuli compared to the no-risk infants. Although conflicting, this evidence indicates that risk condition for language learning impairment affected habituation to speech and non-speech sounds, a finding that did not generalize to the learning of statistical patterns and invariant structures from sequences of visual items in the current study. The visual (vs. auditory) nature of the stimuli we used may well be responsible for the inconsistency between the current and the previous results. Because this is the first study to explore how risk condition for language learning impairment affects visual implicit learning in infants, further studies are needed to expand our understanding of how HR infants allocate attention to and process structured visual sequences.

Despite the lack of group differences in the habituation profile during both the SL and the RL tasks, differences in performance between the TD and HR infants emerged during the post-habituation test phase of the RL task. In the visual SL task, both the TD and the HR infants looked more at the novel than at the familiar test sequences, revealing an intact SL ability. On the contrary, in the visual RL task, only the TD infants discriminated between the novel and the familiar rule at the test, indicating that they were able to extract and generalize the rule learned during the habituation phase. Conversely, the HR infants showed no post-habituation preference for either the novel or the familiar test sequences, suggesting that they either failed at building a stable representation of the familiar rule during habituation, or failed at generalizing the familiar rule to the novel shapes presented during test trials.

Overall, evidence from the TD group replicated previous demonstrations that seven- and eight-months-old infants are sensitive to statistically-based [57] and rule-based [58] structures from sequences of visual items. Evidence from the HR group, on the other hand, added to this earlier evidence by showing, for the first time, that RL abilities, but not SL abilities, are impaired in the presence of familial risk for language learning impairment. Importantly, this finding was further corroborated by the within-subjects comparison performed on the subsample of HR and TD infants who participated in both tasks.

The finding of preserved visual SL abilities in infants at risk for language learning impairment contrasts with previous demonstrations of abnormal SL from speech sequences in children with language and reading problems [37,39], and atypical neural processing of transitional probabilities from visual sequences in developmental dyslexia children [39,41]. Most crucially, our results are at odds with those reported by Kerkhoff et al. [49] with 12-month-old infants at familial risk for developmental dyslexia, who failed to extract the statistical structure embedded in a stream of speech sounds. We believe that discrepancies in the results might likely depend on the nature of the stimuli used in the two tasks, i.e., speech stream in Kerkhoff et al. [49] versus visual stream in the current study. The use of linguistic stimuli might have hindered SL abilities in at-risk infants and individuals with developmental dyslexia/language learning impairment, whose sensitivity to the statistical dependencies hidden in the input might be particularly impacted when they are required to process linguistic sounds. Indeed, in the statistical learning literature, there is evidence of dissociations between statistical learning of linguistic materials versus the learning of non-linguistic materials in typical adult and child learners [69,70]. These are thought to arise from the expectations of the underlying structure that participants have based on their native language experience, which, in the case of language learning impairment individuals, would be defective due to their language deficit. Accordingly, recent studies reported spared visual SL performance in school-aged children with diagnosis of language learning impairment when they were presented with sequences of images [71,72].

Unlike Study 1, HR infants in Study 2 failed to recognize the familiar rule when it was implemented by novel items at test. As HR infants did not differ from TD infants in their habituation performance, one might argue that they were indeed both able to detect and represent the ABA rule presented during habituation, but HR infants could not generalize the familiar rule to the novel test items. Indeed, the lack of discrimination between the familiar ABA rule and the novel ABB rule at test in the HR infants might reflect the difficulty in creating an abstract representation of the rule structure that is independent from the surface features of the stimuli. This interpretation finds support in recent studies showing that 9–11-year-old children with developmental dyslexia learned and recognized visual rule-like patterns in non-transfer tasks where the surface characteristics of the training and the test stimuli remained unchanged, but failed in a transfer task where the stimuli differed but followed the same rules [43,44]. Our data do not allow us to disentangle whether HR infants did not build an abstract representation of the ABA structure during habituation or they did not generalize such structure to the new items at test. Indeed, HR infants in the subsample seemed to show a small trend toward preferring the familiar trials over the novel ones at test. Although this same trend was not present in the whole sample tested in Study 2, it may suggest that HR infants’ encoding of the ABA rule during habituation was insufficient, so that they completed learning during the test. Our data indicate that family-risk infants could not perform a standard visual RL habituation task as efficiently as typically developing infants could.

Our results suggests that SL and RL mechanisms play a distinct role in the development of language learning impairment. As previously discussed, it is known that the ability to extract transitional probability is involved in the acquisition of lexical skills (e.g., [13,14]), and the ability to extract and generalize high-order rules are related to the acquisition of grammar skills (e.g., [20,22,73]). Our results revealed that infants at high risk for language learning impairment succeeded in detecting transitional probability between visual items, while they failed in learning and/or generalizing abstract rules. This finding supports the hypothesis that a mechanism linked to the extraction of grammar structures may contribute to the development of language and reading problems [6,35,49]. This idea is grounded on evidence that school-age children diagnosed with developmental dyslexia and preschool-age children at risk for developmental dyslexia [74,75,76] were deficient in the identification of correct and incorrect morphosyntactic agreement relations, and that children with developmental language disorder showed difficulties in identifying and producing subject-verb agreements (e.g., [77]). Moreover, delays in early syntactic comprehension predict a future diagnosis of developmental language disorder in late talkers (e.g., [2]). These pieces of evidence point to the idea that deficits in children with diagnosis or at familiar risk for language learning impairment are not limited to lexical skills but might be extended to other linguistic domains, like grammar (see also [2,78]). This highlights the importance of including measures of grammar skills in language assessment of children with language learning impairment.

To note, the present findings point to the idea that the ability to detect regularities from the visual input might be helpful to create an effective intervention program based on multimodal stimulation in children with a diagnosis of language learning impairment. For example, it has been shown that infants’ implicit learning is enhanced in the presence of multimodal (visual and auditory) information (e.g., [79]), and that the provision of visual information supports working memory performance in typically developing and language learning impairment children [80]. Therefore, training with regard to the ability to extract structured information from visual (or visual-auditory) streams might support at-risk infants in discovering the auditory patterns embedded in their linguistic environment.

Even though we found evidence of differences between visual SL and RL skills in HR infants, this pattern of results might not generalize to conditions where SL and RL operate on auditory stimuli. To the best of our knowledge, no studies have investigated the efficiency of SL and RL mechanisms in high-risk populations comparing visual vs. auditory stimuli. Future studies are needed to shed light on the extent to which the nature of the stimuli (auditory vs. visual) may constrain implicit learning mechanisms in HR infants.

The present study highlights the potential role of visual implicit learning mechanisms as early markers for atypical language development. Investigating visual implicit learning mechanisms in early stages of development allows us to deepen our understanding of the mechanisms underlying the acquisition of complex abilities like language, regardless of culture, education, and other environmental confounders. The current findings might also have strong implications for the implementation of early intervention programs based on visual learning training in infants at risk for language learning impairment.

## 6. Conclusions

The present findings add to existing evidence of associations between atypical implicit learning abilities and language learning impairment [49] by showing, for the first time, that visual RL, unlike visual SL, is impaired in preverbal infants at family risk for language and learning disorders. Based on this evidence, rather than focusing on implicit learning as a single construct, future research may adopt a multifactorial longitudinal approach to investigate how different learning mechanisms contribute to the developmental trajectory of different aspects of linguistic and communicative abilities, and how tiny variations in the functioning of each learning mechanism relate to different developmental outcomes.

## Figures and Tables

**Table 1:** Descriptive statistics (mean and standard deviations) of demographic characteristics for the TD and the HR infants.

Study 1—Visual SL Task					
	TD (N = 21)	HR (N = 20)			
	M (SD)	M (SD)	t (df)	*p*	Cohen’s d
Gestational weeks	39.06 (1.39)	38.35 (1.63)	1.39 (34)	0.174	0.466
Birth weight (gr)	3270 (413.51)	3192.50 (583.14)	0.448 (34)	0.657	0.150
Bayley Cognitive sub-scales ^1^	11.19 (1.38)	11.78 (1.44)	−1.219 (32)	0.232	−0.419
Mother’s age	32.50 (6.22)	34.65 (3.86)	−1.294 (36)	0.204	−0.421
Father’s age	35.50 (6.07)	36.60 (4.56)	−0.636 (36)	0.529	−0.207
Mother’s educational level ^2^	53.22 (16.65)	56.75 (17.64)	−0.881 (36)	0.423	−0.264
Father’s educational level	54.44 (13.38)	45.00 (18.21)	1.804 (36)	0.080	0.586
Socioeconomic status ^3^	61.11 (16.05)	58.50 (17.78)	0.473 (36)	0.639	0.154

^1^ Cognitive subscale from the Bayley Scales of Infant Development [59]; ^2^ Educational level scored on a 10–90 range scale (10 = elementary school, to 90 = doctoral degree); ^3^ Socioeconomic status scored on the Hollingshead 10–90 range scale (Hollingshead, 1975; 10 = unskilled worker, to 90 = major professional); the higher score among the mother and father was considered.

**Table 2:** Descriptive statistics of the habituation variables (i.e., number of trials, total looking times, and slope of looking times to habituate) for both the TD and HR infants; independent-samples *t*-tests were used to compare habituation performance between the two groups.

Visual SL Task—Habituation Phase					
	TD Infants	HR Infants			
	M (SD)	M (SD)	t (df)	*p*	Cohen’s d
Number of trials	7.10 (1.92)	8.15 (3.66)	1.163 (39)	0.252	0.364
Total looking times (s)	98.74 (61.33)	95.90 (43.31)	0.486 (39)	0.865	0.053
Slope	−3.99 (4.02)	−3.17 (3.48)	0.691 (38)	0.494	0.26

**Table 3:** Descriptive statistics (mean and standard deviation) of demographic characteristics for the TD and the HR infants.

Study 2—Visual RL Task					
	TD (N = 19)	HR (N = 19)			
	M (SD)	M (SD)	t (df)	*p*	Cohen’s d
Gestational weeks	39.35 (1.72)	38.61 (1.88)	1.36 (33)	0.184	0.459
Birth weight (gr)	3361.44 (378.53)	3190.83 (561.14)	1.07 (34)	0.292	0.356
Bayley Cognitive sub-scales	11.22 (1.70)	12.35 (1.12)	−2.312 (33)	0.027 *	−0.782
Mother’s age	33.68 (4.40)	35.58 (3.60)	−1.454 (36)	0.155	−0.472
Father’s age	35.53 (4.09)	37.63 (5.23)	−1.382 (36)	0.175	−0.449
Mother’s educational level	60.56 (13.05)	58.16 (18.50)	0.453 (35)	0.653	0.149
Father’s educational level	53.33 (12.83)	44.74 (19.82)	1.574 (35)	0.126	−512
Socioeconomic status	65.00 (12.00)	61.84 (15.48)	0.691 (35)	0.494	0.227

* significant differences between the two groups.

**Table 4:** Descriptive statistics and *t*-test results comparing habituation performance (i.e., number of trials, total looking times, and slope) between TD and HR infants.

Visual RL Task—Habituation Phase					
	TD Infants	HR Infants			
	M (SD)	M (SD)	t (df)	*p*	Cohen’s d
Numbers of trials	9.05 (3.55)	9.05 (4.59)	0.000 (36)	1.00	4.102
Total looking times (s)	65.23 (37.79)	64.76 (38.14)	0.038 (36)	0.970	0.012
Slope	1.30 (1.04)	−1.54 (1.28)	0.640 (36)	0.526	0.208

**Table 5:** Descriptive statistics (mean and standard deviations) of demographic characteristics for the TD and HR subsamples of infants who participated in both tasks. The two groups are homogeneous on all considered variables.

Longitudinal Sample					
	TD (N = 10)	HR (N = 15)			
	M (SD)	M (SD)	t (df)	*p*	Cohen’s d
Gestational weeks	39.50 (1.35)	38.27 (1.83)	1.82 (23)	0.082	0.743
Birth weight (gr)	3361.50 (456.97)	3141.00 (601.71)	0.983 (23)	0.336	0.401
Bayley Cognitive sub-scales	11.10 (1.66)	12.15 (1.21)	−1.759 (21)	0.093	−0.740
Mother’s age	33.50 (5.38)	35.33 (3.77)	−1.004 (23)	0.326	−0.410
Father’s age	35.70 (3.23)	37.00 (4.65)	−0.767 (23)	0.451	−0.313
Mother’s educational level	56.00 (13.50)	59.67 (17.53)	−0.558 (23)	0.582	−0.228
Father’s educational level	54.00 (16.46)	45.33 (19.95)	1.137 (23)	0.267	0.464
Socioeconomic status	63.00 (14.94)	61.33 (16.74)	0.254 (23)	0.802	0.104

## Data Availability

The data presented in the study are available on request from the corresponding author. The data are not publicly available due to privacy.
